# Versatility of use of fibrin glue in wound closure and vitreo-retinal surgery

**DOI:** 10.1186/s40942-021-00298-5

**Published:** 2021-04-15

**Authors:** Gabriela Lopezcarasa-Hernandez, Jose-Francisco Perez-Vazquez, Jose-Luis Guerrero-Naranjo, Maria A. Martinez-Castellanos

**Affiliations:** 1 Retina and Vitreus Department, Asociacion para Evitar la Ceguera en México, IAP, Vicente García Torres 46, Col. Barrio San Lucas, Coyoacan, 04030 Mexico City, Mexico; 2 Hospital Angeles Lomas, Mexico City, Mexico

**Keywords:** Fibrin glue, Vitreoretinal surgery, Retinal detachment surgery, Rhegmatogenous retinal detachment

## Abstract

**Background:**

Fibrin glue is an absorbable blood-derived product, a biological tissue adhesive which imitates the final stages of the coagulation cascade, it produces a firm clot, forming a seal along the whole length of the wound, the resultant fibrin clot degrades physiologically into granulation tissue 2 weeks after the application. Biological glue has been used extensively in many forms of surgical procedures. Its use in eye surgery has grown lately as we have evidence that showed it was effective in securing conjunctival grafts in pterygium surgery, in securing wounds after glaucoma surgery and more importantly in 20G and 23G vitrectomy.

**Purpose:**

The aim of this study is to present our experience in the use of fibrin glue in vitreoretinal surgery.

**Material and methods:**

We included 281 eyes of 221 patients who underwent vitreoretinal surgery during the period of May 2009 to July 2012, the preoperative diagnoses were as following: proliferative diabetic retinopathy, rhegmatogenous retinal detachment, macular hole, epiretinal membrane, luxation of cataratous nucleous and cortex, intraocular lens luxation, penetrating trauma, silicone extraction, phaco + IOL + vitrectomy + Ahmed valve implant, vitreous biopsy and optic nerve pit associated to macular detachment. The procedures were performed with Alcon Accurus Surgical System 20-gauge, 23-gauge or a combination of both. We used fibrin glue in all of the 20-gauge sclerotomies and leaking 23-gauge sclerotomies, scleral wound for IOL extraction, conjunctival peritomy for buckle implantation, conjunctiva in Ahmed valve implant, corneal graft in corneal perforation in trauma and leaking corneal wounds for phacoemulsification, in an optic pit, and in subretinal space in a giant retinal tear.

**Results:**

We did not use any suture in any of the patients throughout the different procedures, there was no leakage in any wounds in the postoperative period, we found no inflammatory reaction, infection, and whenever we had excess amount, it was trimmed. Two patients presented a small dehiscence of the wound that was corrected in-office with a small amount of fibrin glue in the post-operative period.

**Conclusions:**

Fibrin glue reduces surgical time, it is a good sealant, safe, with minimal allergic or toxic reactions and inflammation, minimizes bleeding, easy to undo and that eventually degrades. This small series shows that fibrin glue is a viable alternative for tissue coaptation in vitreoretinal surgery. However, further studies are required before fibrin glue takes the place of sutures.

## Background

The new advances in vitreoretinal surgery have the tendency to switch to sutureless surgery in the aim of saving surgical time and diminishing the inflammation associated to sutures. A number of recent developments have established tissue adhesives as attractive alternatives to sutures. Currently, fibrin glue is being used for conjunctival closure following pterygium and strabismus surgery, lamellar corneal grafting, closure of corneal perforations and management of conjunctival wound leaks after trabeculectomy [1]. There is a limited number of reports of the use of tissue adhesives in vitreoretinal surgery as early as 1988, Zauberman et al. have reported its use for conjunctival wound closure following retinal detachment surgery [2]. Mentens compared the efficacy of fibrin glue in comparison with conjunctival closure by sutures following 20-gauge needle pars plana vitrectomy in 504 eyes [3, 4]. In these publications the authors reported that fibrin glue offers significantly better results than suturing for closure of conjunctival wounds. In another study, Batman et al. supported the view of Mentens and suggested that in case of persistence of leaking wound following transconjunctival-sutureless vitreoretinal surgery, application of fibrin glue is a better alternative over suturing [5]. Fibrin glue has other intraoperative applications during vitreoretinal surgeries as well. It has been found to not have any toxic effects on retinal function or structure in a rabbit model [6]. Its use has also been reported in macular hole surgery, and has been described recently in the management of optic disc pit-associated macular detachments [7–9]. Fibrin glue has also been used to stabilize keratoprosthetic devices during vitreoretinal surgeries [10].

The latest literature supports procedures such as glue-assisted retinopexy for rhegmatogenous retinal detachment (GuARD). Anatomical success can be archived in retinopexy procedures without the use of gas or silicon-oil tamponade. Advantages such as no need for maintaining a specific position during the post-operative period. However, a closer follow up is need due to suspected epiretinal proliferation [11].

Fibrin glue is a biological tissue adhesive which imitates the final stages of the coagulation cascade when a solution of human fibrinogen is activated by thrombin (the two components of fibrin glue). Fibrin glue includes a fibrinogen component and a thrombin component, both prepared by processing plasma. It can be prepared from the patients’ own blood or obtained as a commercially available preparation. Fibrin glue produces a firm clot, forming a seal among the whole length of the wound, the resultant fibrin clot degrades physiologically after granulation tissue two weeks after the application [12–14].

The aim of this study is to present our experience in the use of fibrin glue in wound closure and vitreoretinal surgery.

## Methods

In our study, patient enrollment was performed in a consecutive manner, every patient with surgical indication for retinopexy and vitrectomy was considered for use of fibrin glue within their procedure.

**Fig. 1:**
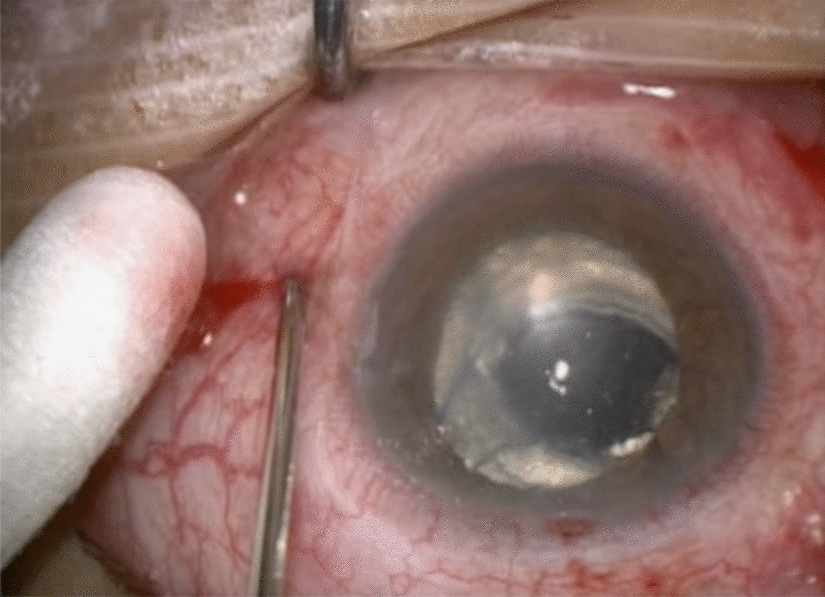
Use of fibrin glue to close a leaking sclerotomy using a 20-gauge cannula

**Fig. 2:**
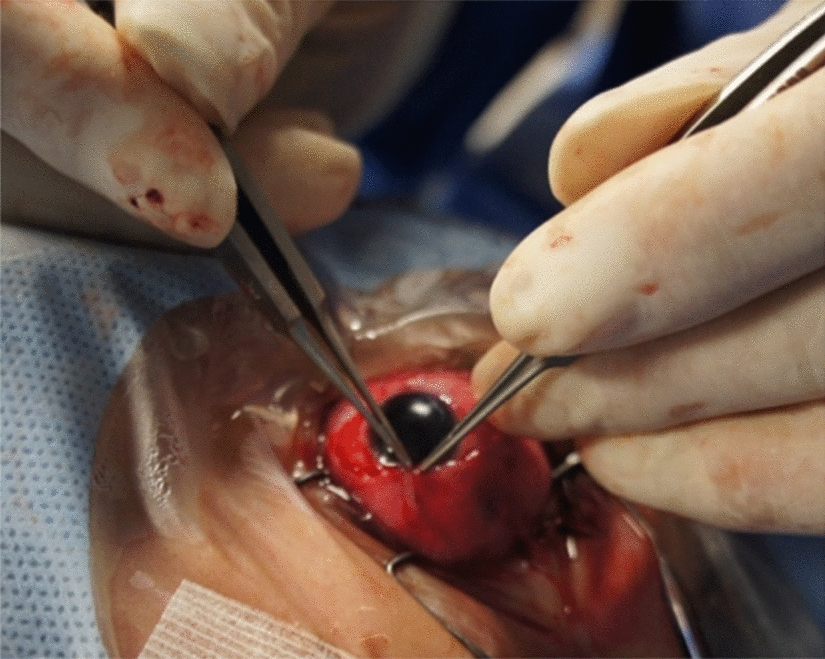
Fixation of conjunctiva after applying fibrin glue in its borders for 60 s

**Fig. 3:**
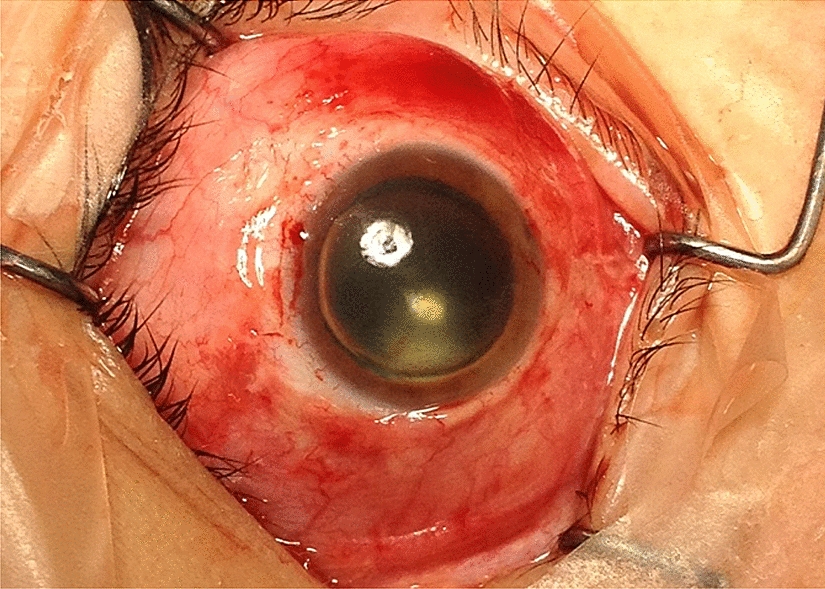
Use of fibrin glue in conjunctival peritomy fixation after a successful retinal detachment surgery. Fibrin glue was used in inferior and temporal conjuntival incisions

The procedures were performed with Accurus Surgical System (Alcon Surgical, Forth Worth, TX, USA) at 20-gauge or 23-gauge. We used fibrin glue in all of the 20-gauge sclerotomies and leaking 23-gauge sclerotomies, (Fig. 1) scleral wound for IOL extraction, conjunctival peritomy for buckle implantation (Figs. 2, 3), conjunctiva in Ahmed valve implant, corneal graft in corneal perforation in trauma and corneal wounds for phacoemulsification, in an optic nerve pit, and in subretinal space in giant retinal tear to prevent slippage.

### Technique for application

**Fig. 4:**
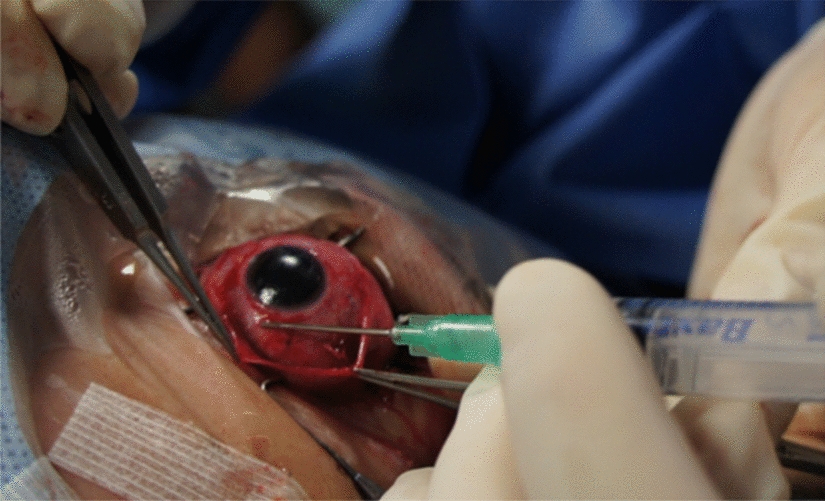
Application of fibrin glue during a retinopexy procedure using a 20-gauge cannula and a 3 ml syringe

The two components of fibrin glue were applied sequentially to prevent clotting within the needle. We used both components loaded into two syringes. The thrombin is first applied on the area of interest, followed by a thin layer of fibrinogen. In a minute or two, coagulation starts and by two or three minutes, polymerization is complete. The physiological final pathway of coagulation is replicated. Factor XIII (present in the fibrinogen component of the glue) cross links and stabilizes the clot's fibrin monomers while aprotinin inhibits fibrinolytic enzymes, consequently resulting in a stable clot. The tissue is pressed gently over the glue for 60 s for firm adhesion (Fig. 4). At the end of the procedure, pad and bandage is applied after instillation of steroid and antibiotic drops. The commercially available form of fibrin glue we used comes installed with double-barreled injection system, but due to lack of control over the mixing time of both injection components and the risk of a sudden burst of pressure when congealed glue frees outside the cannula, and risk of tissue damage, we opted out of the pre-installed system. Instead, we layered both components separately while at the same time holding both tissue borders together, until visible fixation was obtained.

### Vitrectomy

Our use of fibrin glue during vitrectomy surgery for different diagnoses consisted of sealing sclerotomies whether they were leaking or not (Fig. 1). A thin layer of thrombin was applied around the sclerotomy borders and soon after, using curved forceps, pressure was applied to close the borders, then, a thin layer of fibrinogen was applied right after tissue repositioning was made to seal the port of entrance.

## Results

We included 281 eyes of 221 patients who underwent vitreoretinal surgery during a 10-year period (Table 1). The preoperative diagnoses were as following: proliferative diabetic retinopathy, rhegmatogenous retinal detachment, macular hole, epiretinal membrane, luxation of cataratous nucleous and cortex, intraocular lens luxation, penetrating trauma, silicone extraction, phaco + IOL + vitrectomy + ahmed valve implant, vitreous biopsy and optic nerve pit associated to macular detachment.

**Table 1:** Study procedures

Procedure	Number of patients	Number of eyes	Tamponade	Gauge
Proliferative diabetic retinopathy (either vitrectomy or combined surgery—phacoemulsification, implant of intraocular lens and vitrectomy)	86	142	SO 1000cs-46 SO 5000cs-48 SF6 gas-32 C3F8 gas-1 Air-4 BSS-11	20G-93 23G-49
Regmatogenus retinal detachment (either vitrectomy or combined surgery—phacoemulsification, intraocular lens implant and vitrectomy or lensectomy and vitrectomy)	35	36	SO 1000cs-8 SO 5000cs-6 SF6 gas-14 C3F8 gas-5 Heavy silicon oil-3	20G-15 23G-20
Macular hole (either vitrectomy or combined surgery—phacoemulsification, intraocular lens implant and vitrectomy)	28	28	SF6 gas-28	20G-11 23G-17
Epiretinal membrane (either vitrectomy or combined surgery—phacoemulsification, intraocular lens implant and vitrectomy)	18	19	Air-8 BSS-6 SF6-5	20G-12 23G-7
Silicon extraction	15	15	BSS-12 Air-3 Silicon oil 5000cs-1	20 G-15
Nucleus luxation	11	11	BSS-9 Air-2	20 G-8 23 G-3
Luxation of intraocular lens	9	9	BSS-7 Air-1 SO 5000cs-1	20G-8 23G-1
Penetrating trauma Intraocular foreign body (4)	6	6	SO 5000cs-3 SF6 gas-1 C3F8 gas-1 Air-1	20G 20
Combined surgery (cataract phacoemulsification + vitrectomy + Ahmed valve implantation)	3	5	SO 1000cs-2 SO5000cs-1 BSS-2	20G-4 23G-1
Macular detachment associated to optic pit	1	1	C3F8 gas-1	23G
Vitreous biopsy	1	1	BSS-1	23G
Giant retinal tear	1	1	SO1000cs	20G

*SO* silicon oil, *BSS* balanced-saline solution, *SF6* sulfur hexafluoride, *C3F8* octafluoropropane, *CS* centistokes, *G* gauge

## Discussion

In this study, we aimed to describe the different applications of fibrin glue within vitreoretinal surgery. Throughout the development of our experience with fibrin glue, we noted several advantages that we will further discuss. This material is blood-derived, meaning it has a biological origin as first described by Tassman in 1950, who proposed its first application in ophthalmologic surgery mainly to unite tissues and eliminate the use of sutures [15]. We used commercially available fibrin glue in all our procedures due to its ease of use, however, as described by Bhatia et al., fibrin glue can be obtained by the recipient’s own blood, using cryoprecipitate, but low concentrations of fibrinogen (2–4 mg/ml) require large amounts of blood donated to obtain a very small amount of viable fibrin glue. These two limitants discourage the use of recipient-derived fibrin glue preparations [16].

One advantage in the use of fibrin glue is the absence of suture removal procedure in the post-operative period since it has been previously described the risk of endophthalmitis following suture removal [17]. Due to its plasticity and biodegradability it is very easy to use and it’s safe to manipulate in the postoperative period at the office, using the slit lamp.

**Fig. 5:**
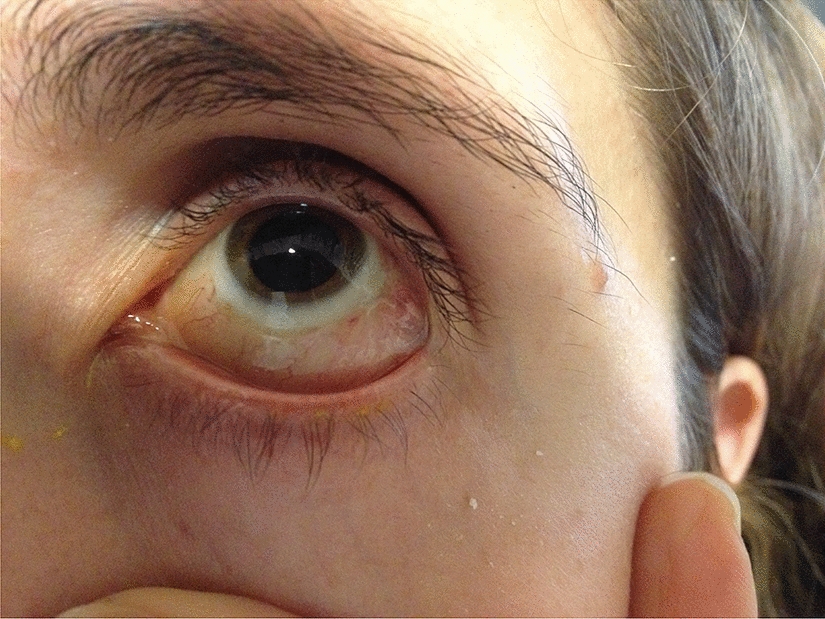
Follow-up of a patient after use of fibrin glue to fixate the nasal conjuctival incision. On the temporal conjuctiva a granuloma was formed secondary to a placement of a 7–0 Vicryl suture. It’s evident the greater inflammation that sutures cause to the conjunctiva at 4 weeks follow-up, compared to fibrin glue in the nasal conjunctiva

Uy et al. described a decreased inflammatory tissue reaction, following pterygium surgery, which in our experience is compatible with less inflammatory reaction after using fibrin glue to seal a leaking sclerotomy or conjunctival fixation after peritomy for PPV (Fig. 5) [18]. Less inflammatory reaction provides the patient with more comfort after the procedure, something we have related to a better outcome.

There are several publications referring to the use of fibrin glue within wound closure in external procedures, however there is a limited number of evidence regarding its use in vitreoretinal surgery both internally and externally.

Since fibrin glue is a blood-derived product there are reports of its safety in the use within intraocular tissues without severe toxic reactions. Historically, the use of blood clots in management of macular hole surgery has been a coadyuvant technique for closure of macular hole, without toxic or adverse events reported with the eye. This past experience encourages the use of blood-derived products like fibrin glue as a safe tool for wound closure and application in vitreo retinal surgery [19].

## Conclusions

In our experience, fibrin glue may be useful in reducing surgical time as it is a good sealant, safe, with minimal allergic or toxic reactions and inflammation, minimizes bleeding, easy to undo and that eventually disappears. This series shows us that fibrin glue is a viable alternative for tissue coaptation in vitreoretinal surgery. However, further and larger studies are required. A prospective point of view should be necessary to completely evaluate the fibrin glue role in the post-operative period. Comparative, randomized trials would be of great help to fully contrast fibrin glue vs conventional surgical techniques. Different surgical techniques in vitreoretinal diseases can be evaluated to fully exploit fibrin’s glue potential to aid in the outcome of patients’ procedures.

## Data Availability

The data that support the findings of this study are available on request from the corresponding author, [GLH]. The data are not publicly available due to restrictions in medical history access in the hospitals.
